# Anti-Diabetic Nephropathy Activities of Polysaccharides Obtained from *Termitornyces albuminosus* via Regulation of NF-κB Signaling in db/db Mice

**DOI:** 10.3390/ijms20205205

**Published:** 2019-10-21

**Authors:** Chang Yang, Qi Feng, Huan Liao, Xinlei Yu, Yang Liu, Di Wang

**Affiliations:** 1 Engineering Research Center of Chinese Ministry of Education for Edible and Medicinal Fungi, Jilin Agricultural University, Changchun 130118, China; yangchang1316@mails.jlu.edu.cn; 2 School of Life Sciences, Jilin University, Changchun 130012, China; fengqi1316@mails.jlu.edu.cn (Q.F.); liaohuan1316@mails.jlu.edu.cn (H.L.); yuxl1316@mails.jlu.edu.cn (X.Y.)

**Keywords:** Type 2 diabetes mellitus, diabetic nephropathy, *Termitornyces albuminosus*, polysaccharides, NF-κB

## Abstract

*Termitornyces albuminosus* is a kind of traditional Chinese edible fungus rich in nutrients and medicinal ingredients, and it has anti-oxidative, analgesic and anti-inflammatory effects. However, the hypoglycemic and nephroprotective effects of polysaccharides separated from *T. albuminosus* (PTA) have not been reported. The properties of PTA were analyzed in a BKS.Cg-Dock7^m^ +/+ Lepr^db^/JNju (db/db) mouse model of diabetes. After the administration of PTA for eight weeks, the hypoglycemic and hypolipidemic activities of PTA in the db/db mice were assessed. The results of a cytokine array combined with an enzyme-linked immunosorbent assay confirmed the anti-oxidative and anti-inflammatory activities of PTA. An eight-week administration of PTA caused hypoglycemic and hypolipidemic functioning, as indicated by suppressed plasma glucose levels, as well as the modulation of several cytokines related to glycometabolism, in the sera and kidneys of the mice. PTA treatment also had a protective effect on renal function, restoring renal structures and regulating potential indicators of nephropathy. In the kidneys of the db/db mice, PTA treatment reduced the activation of protein kinase B, the inhibitor of κB kinase alpha and beta, and the inhibitor of κB alpha and nuclear factor-κB (NF-κB). We establish the hypoglycemic, hypolipidemic, and anti-diabetic nephropathy effects of PTA, and we find that the renal protection effects of PTA may be related to anti-inflammatory activity via the regulation of NF-κB signaling.

## 1. Introduction

Diabetes mellitus (DM), a chronic endocrine and metabolic disease, affects patients by causing immune dysfunction, the generation of damaging free radicals, and inflammatory injury [1]. In 2017, nearly 114 million Chinese people suffered from DM, the highest number of patients in any country worldwide [2]. High levels of blood glucose (hyperglycemia) in DM patients lead to the lipid metabolism disorder hypertriglyceridemia, which is responsible for insulin resistance [3]. Due to sustained hyperglycemia, about 20%–40% of patients with type 1 or type 2 DM develop diabetic nephropathy (DN) [4], which affects about 21.3% of all DM patients in China [5]. End-stage renal failure caused by DN is now the main cause of death in 40%–50% of diabetic patients [6].

Though the pathogenesis of DN is complex, several studies have found that oxidative stress plays a key role in the occurrence and development of DN [7]. According to the “unified theory of diabetes,” hyperglycemia helps to produce excessive reactive oxygen species (ROS), which activate multiple signaling pathways responsible for diabetic complications, including DN [8]. However, due to disorders of glucose metabolism and hemodynamic abnormalities, inflammation is also a key factor for the sustained development of DN [9,10]. Under hyperglycemic conditions, the innate immune cells of the kidney produce tumor necrosis factor-α (TNF-α), interleukins (ILs), monocyte chemotactic protein (MCP-1), and other inflammatory factors that expand the inflammatory response [11]. 

Currently, the main treatment strategy for DN is to control blood glucose, blood pressure, and lipids by using anti-oxidants and glycosylation end-product inhibitors [12,13]. Patients suffering from end-stage DN must be treated with renal dialysis and kidney transplantation [14,15]. The existing treatment options are expensive and cause various side effects including irritating anorexia and liver and kidney toxicity [16]. Therefore, systematic research of the molecular mechanisms of DN is essential to effectively control the occurrence and development of DN in DM patients.

Polysaccharides obtained from fungi such as *Suillellus luridus* [17], *Amillariella mellea* [18] and *Dictyophora indusiate* [19] have been reported to show anti-diabetic activities and improve renal function. Polysaccharides isolated from *Inonotus obliquus* [20] and *Tuber melanosporum* [21] show anti-diabetic and anti-nephritic activities via the modulation of oxidative stress. Purified polysaccharides from *Irpex lacteus* inhibit the process of membranous glomerulonephritis via the regulation of the nuclear factor-κB (NF-κB) pathway [22]. *Termitornyces albuminosus* is an edible fungus mainly found in Africa and Asia, and it is especially distributed in the Guizhou province of China [23]. *T. albuminosus* is rich in nutrients and various medicinal ingredients including polysaccharides, saponins and polyphenols [24]. Previous studies have shown that *T. albuminosus* has anti-oxidative, [25], analgesic, and anti-inflammatory effects [26]. However, there has been no direct research on any hypoglycemic or anti-diabetic nephropathic effects of *T. albuminosus* and its polysaccharides. 

In this study, we purified polysaccharides from *T. albuminosus* (PTA) fruiting bodies and then characterized their structures. In BKS.Cg-Dock7^m^ +/+ Lepr^db^/JNju (db/db) mice, we observed hypoglycemic, hypolipidemic, and anti-diabetic nephropathic effects of PTA. Further, we found that PTA-mediated renal protection under hyperglycemic conditions was related to the regulation of inflammatory cytokines via NF-κB signaling. 

## 2. Results

### 2.1. Characterization of PTA 

**Figure 1:**
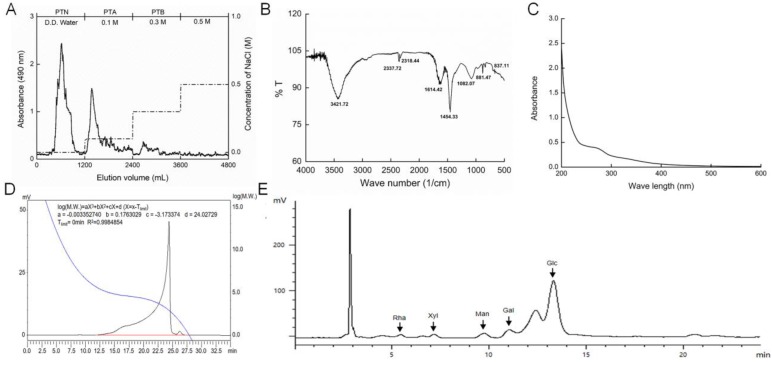
Purification and characterization of polysaccharides from *Termitornyces albuminosus* (PTA). (**A**) Crude polysaccharides were isolated using diethylethanolamine-52 (DEAE-52), and PTA was obtained by elution using 0.1 M NaCl. (**B**) Fourier transform infrared spectroscopy spectrum of PTA. (**C**) UV spectrum of PTA. (**D**) Molecular weight analysis using a high performance liquid chromatography (HPLC) system equipped with a TSK-GEL G4000PWXL column. (**E**) Analysis of monosaccharides composition after acid hydrolyzing of PTA with a HPLC system.

The purification curve of PTA via diethylethanolamine-52 (DEAE-52) is shown in Figure 1A. In the Fourier transform infrared (FT-IR) spectra, the broad band around 3422 cm^−1^ represents the characteristic peak of hydrogen bonded O–H stretching vibrations. The signals around 1614 and 1454 cm^−1^ designate the asymmetric and symmetric stretching, respectively, of carboxylate anion groups (C–O). The absorption at 1082 cm^−1^ indicates the existence of a pyranose unit (Figure 1B). Few nucleic acids or proteins were found in the PTA samples (Figure 1C). We calculated the molecular weight of PTA as 11.649 kDa (Figure 1D). The main monosaccharide content in PTA was D-glucose (Glc), with small quantities of D-galactose (Gal), D-mannose (Man), L-rhamnose (Rha), and D-xylose (Xyl) (Figure 1E). 

The hypoglycemic activity of PTA was tested by the detection of fasting blood glucose levels, which were strongly reduced in PTA-treated mice beginning from the second week *(p <* 0.05, Table A1). In addition, both metformin (Met) and PTA influenced the bodyweight of the mice (*p* < 0.05) (Table A1).

**Figure 2:**
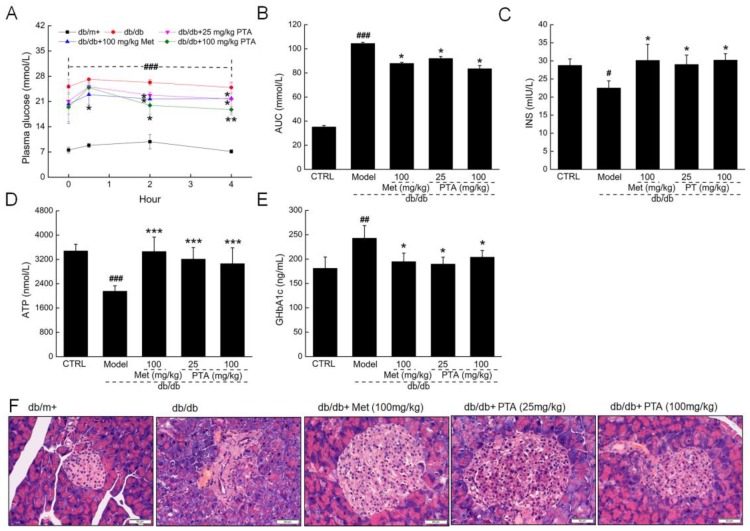
The hypoglycemic effects of PTA on db/db mice. Blood glucose levels (**A**) and the values of area under the curve (AUC) (**B**) within 4 h after glucose administration. ELISA detection of insulin (**C**), ATP (**D**), and glycosylated hemoglobin A1c (GHbA1c (**E**) levels in the sera of db/db mice. Data are expressed as means ± SD (n = 12). ^#^ *p* < 0.05, ^##^ *p* < 0.01 and ^###^ *p* < 0.001 vs. C57BLKS/J-LepR^db/+^ (db/m^+^) mice, * *p* < 0.05, ** *p* < 0.01 and *** *p* < 0.001 vs. non-treated db/db mice. (**F**) Histopathological analysis of pancreatic islets via hematoxylin and eosin (H and E) staining (scale bar: 50 μm; magnification: 200×) (n = 6).

Patients with diabetes show problems with glucose utilization and metabolism due to a lack of insulin secretion [27]. In addition to fasting blood glucose levels, the oral glucose tolerance test (OGTT) is another common method for assessing systemic glucose tolerance. Compared with the untreated db/db mice, PTA-treated mice showed a significant improvement in blood glucose metabolism, with reduced blood glucose levels two hours after glucose gavage (*p* < 0.05, Figure 2A) and a reduced area under the curve of blood glucose at different time points (*p* < 0.05, Figure 2B). The administration of PTA for eight weeks resulted in a 28.9% increase in insulin levels (*p* < 0.05, Figure 2C) and a 42.1% increase adenosine triphosphate (ATP) levels in the db/db mice (*p* < 0.001, Figure 2D). 

Met and PTA strongly reduced the serum levels of glycosylated hemoglobin A1c (GHbA1c) (*p* < 0.05, Figure 2E), suggesting that PTA can promote insulin (INS) secretion and restore blood sugar metabolism. 

Islet hypertrophy, an increased number of islet cells, and the degeneration of islet B cell vacuoles were noted in the pancreases of db/db mice when compared with db/m^+^ mice, and all three markers were reduced after eight-week PTA and Met treatment (Figure 2F).

### 2.2. PTA Regulates Lipid Metabolism in db/db Mice 

**Figure 3:**
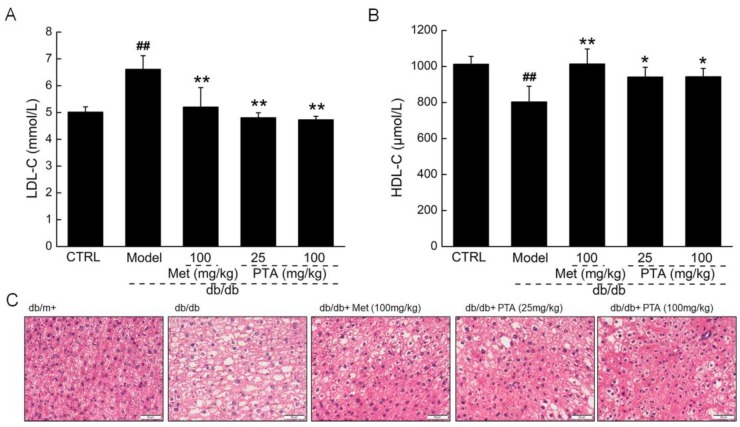
PTA regulates lipid metabolism in db/db mice. PTA reduced the levels of (**A**) low density lipid cholesterol (LDL-C and enhanced the levels of (**B**) high density lipid cholesterol (HDL-C in sera of db/db mice after eight-week administration. Data are expressed as means ± SD (n = 12). ^##^ *p* < 0.01 vs. db/m^+^ mice, * *p* < 0.05 and ** *p* < 0.01 vs. non-treated db/db mice. (**C**) Histopathological analysis of liver via H and E staining (scale bar: 50 μm) (magnification: 200×) (n = 6).

In Type 2 DM (T2DM), the disorder of lipid metabolism induced by hyperglycemia can cause serious obesity and complications [28]. High density lipid cholesterol (HDL-C) can promote the flow of total cholesterol (TC) and triglyceride (TG) from peripheral tissues to the liver for metabolism; meanwhile, excess low density lipid cholesterol (LDL-C) can form atherosclerotic plaque lesions that deposit on blood vessel walls [29]. PTA treatment strongly reduced the levels of LDL-C (*p* < 0.01, Figure 3A) and enhanced the levels of HDL-C (*p* < 0.05, Figure 3B) in the sera of db/db mice. Due to the disorder of fat metabolism in T2DM, hepatic cells are frequently in a state of fatty degeneration and filled with lipid droplets of varying sizes. These characteristics were noted in db/db mice and were strongly reduced after PTA administration for eight weeks (Figure 3C).

### 2.3. Renal Protection by PTA in db/db Mice 

**Figure 4:**
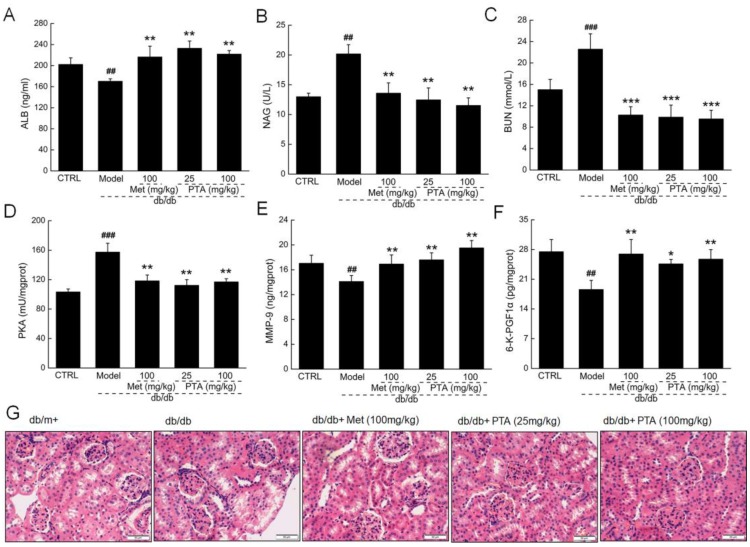
Renal-protective effects of PTA in db/db mice after eight-week administration. PTA enhanced the levels of (**A**) albumin (ALB) and reduced the levels of (**B**) N-acetyl-β-D-glucosidase (NAG) and (**C**) blood urea nitrogen (BUN) in sera of db/db mice. PTA reduced the levels of (**D**) cAMP-dependent protein kinase (PKA) and enhanced the levels of (**E**) matrix metalloproteinase-9 (MMP-9) and (**F**) 6-keto prostaglandin F 1α (6-K-PGF1α) in kidneys of db/db mice. Data are expressed as means ± SD (n = 12). ^##^ *p* < 0.01 and ^###^ *p* < 0.001 vs. db/m^+^ mice, * p < 0.05, ** p < 0.01 and *** p < 0.001 vs. non-treated db/db mice. (**G**) Histopathological analysis of kidney via H and E staining (scale bar: 50 μm) (magnification: 200×) (n = 6).

DN is one of the most serious life-threatening complications in T2DM. The administration of PTA showed significant renal protection, as indicated by the increased serum levels of albumin (ALB) (*p* < 0.01, Figure 4A), increased renal levels of matrix metalloproteinase-9 (MMP-9) (*p* < 0.01, Figure 4E) and 6-keto prostaglandin F 1α (6-K-PGF1α) (*p* < 0.05, Figure 4F), and reductions in urine levels of N-acetyl-β-D-glucosidase (NAG) (*p* < 0.01, Figure 4B), serum levels of blood urea nitrogen (BUN) (*p* < 0.001, Figure 4C), and renal levels of cAMP-dependent protein kinase (PKA) (*p* < 0.01, Figure 4D) in the PTA-treated db/db mice. Moreover, the histology showed that glomerular mesangial hyperplasia, the thickening of the vascular basement membrane, and the slight leakage of plasma protein in the renal capsule were all reduced after PTA administration when compared to untreated db/db mice (Figure 4G).

### 2.4. Anti-Inflammation and Anti-Oxidation Pathways are Involved in PTA-Mediated Renal Protection 

**Figure 5:**
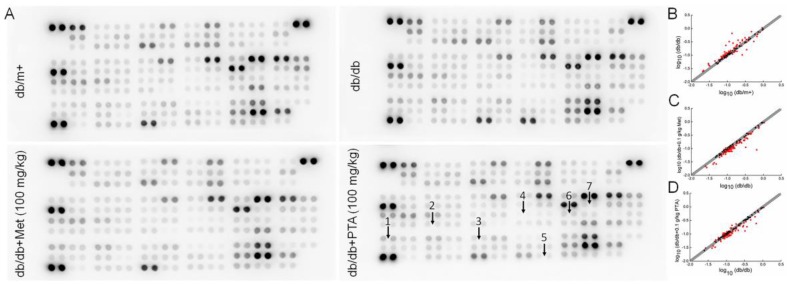
Analysis of inflammatory factors expressed in the kidneys of db/db mice using a Proteome Profiler Mouse XL Cytokine Array. (**A**) The graphical representation of cytokine expressions. (**B**–**D**) Scatter diagram of 111 different cytokines (n = 3). The relative density represents the ratio of the absolute value and the reference spot value. Red dots indicate factors with a change of >10%. (**B**) db/db mice vs. db/m^+^ mice, (**C**) db/db mice vs. Met (metformin)-treated db/db mice and (**D**) db/db mice vs. PTA-treated db/db mice. 1, leptin (LEP); 2, interlekin-12 (IL-12); 3, macrophage colony-stimulating factor (M-CSF); 4, IL-2; 5, tumor necrosis factor-α (TNF-α); 6, IL-4; and 7, interferon-γ (IFN-γ).

The changes of 111 types of cytokine are provided in Table A2. Compared with the vehicle-treated db/db mice, 32 types of cytokine were reduced by 10% or more, and five types of cytokines were increased by 10% or more in PTA-treated db/db mice relative to untreated mice (Figure 5). Based on these results, seven pro- and anti-inflammation cytokines were further analyzed using an enzyme-linked immunosorbent assay (ELISA). Compared with vehicle-treated db/db mice, eight-week PTA administration resulted in reductions of >17.96% (*p* < 0.05), 22.22% (*p* < 0.01), >22.82% (*p* < 0.01), >20.96% (*p* < 0.01), >20.08% (*p* < 0.01) in the renal levels of IL-2, IL-12, TNF-α, interferon-γ (IFN-γ) and macrophage colony-stimulating factor (M-CSF), respectively, as well as >16.67% (*p* < 0.01) and >75.66% (*p* < 0.001) increases in the renal levels of IL-4 and leptin (LEP), respectively (Table 1). Met did not affect the levels of TNF-α of db/db mice (Table 1).

**Figure A1:**
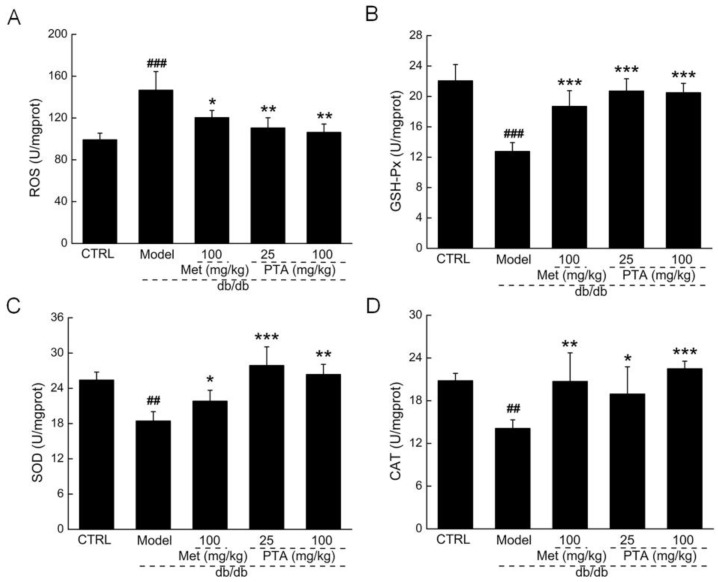
Effects of PTA on oxidative stress-related factors in the kidneys of db/db mice. PTA enhanced the levels of (**A**) reactive oxygen species (ROS), and it reduced (**B**) glutathione peroxide (GSH-Px), (**C**) superoxide dismutase (SOD) and (**D**) catalase (CAT) in the kidneys of db/db mice, as detected via ELISA. Data are presented as means ± SD (n = 12). ^##^ *p* < 0.01 and ^###^ *p* < 0.001 vs. db/m^+^ mice, * *p* *<* 0.05, ** *p* < 0.01 and *** *p* < 0.001 vs. non-treated db/db mice.

Oxidative stress plays a key role in the development and progression of DN [30]. Compared with vehicle-treated db/db mice, PTA administration strongly reduced the levels of ROS (*p* < 0.01, Figure A1A) and enhanced the levels of glutathione peroxide (GSH-Px) (*p* < 0.001, Figure A1B), superoxide dismutase (SOD) (*p* < 0.01, Figure A1C) and catalase (CAT) (*p* < 0.05, Figure A1D) in the kidney. This suggests that PTA has the capacity to scavenge oxygen-free radicals.

### 2.5. NF-κB Signaling Might be Involved in PTA-Mediated Renal Protection 

**Figure 6:**
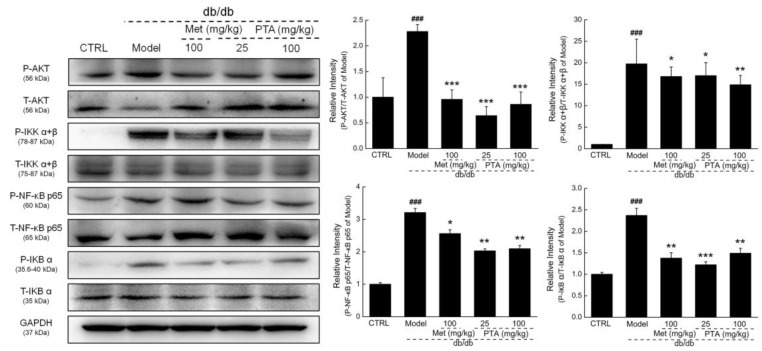
Effects of PTA on nuclear factor-κB (NF-κB) signaling in the kidneys of db/db mice. PTA reduced the phosphorylation levels of protein kinase B (AKT), inhibitor of κB kinase alpha + beta (IKKα+β), inhibitor of κB alpha (IκBα) and NF-κB p65 in the kidneys of db/db mice. Data are expressed as means ± SD (n = 6). ^###^ *p* < 0.001 vs. db/m^+^ mice, * *p* < 0.05, ** *p* < 0.01 and *** *p* < 0.001 vs. non-treated db/db mice.

Compared with vehicle-treated db/db mice, Met and PTA strongly reduced the phosphorylation levels of protein kinase B (AKT) (*p* < 0.001), inhibitor of κB kinase alpha + beta (IKKα+β) (*p* < 0.05), inhibitor of κB alpha (IκBα) (*p* < 0.01), and NF-κB (*p* < 0.01) in the kidneys of db/db mice (Figure 6). 

## 3. Discussion

We analyzed polysaccharides from *T. albuminosus* fruiting bodies, and we characterized them with respect to hypoglycemic, hypolipidemic, and anti-diabetic nephropathic activities in the db/db mouse model of DM. The hypoglycemic activity of PTA is indicated by its suppression of fasting blood glucose levels, enhancement of glucose tolerance, and recovery in serum levels of GHbA1c and insulin. Hyperglycemia causes an increase in protein glycosylation, leading to an increase of glycated hemoglobin [31]. Once the oxygen carrying capacity of hemoglobin is reduced, the over-production of ROS results and is responsible for organ hypoxia in patients with diabetes [32]. The enhanced production and reduced clearance rate of ROS causes an imbalance between anti- and pro-oxidation, which is considered to be the leading factor of the occurrence of oxidative stress [33]. Oxidative stress plays an important role in the pathological process of T2DM. SOD, an anti-oxidant enzyme, has been reported as the first barrier for scavenging free radicals [34], which were strongly enhanced after PTA administration in the kidney tissues of db/db mice. In addition, oxidative stress abolishes the normal function of β-cells and induces the generation of pro-inflammatory mediators, further causing inflammation in islets [35].

The glucose disposal and glycogen accumulation stimulated by insulin is an important way of regulating glucose concentration [36]. During the process of diabetes, the role of glycolysis is weakened, gluconeogenesis is enhanced, and glycogen synthesis disorder is observed [37]. Gluconeogenesis in DM patients causes hyperglycemia, which is relevant to insulin resistance and dyslipidemia [38]. Insufficient insulin also leads to the accumulation of lipids in diabetic patients. The dramatic changes in plasma lipid and lipoprotein profiles caused by hyperglycemia have been reported as risk factors for nonalcoholic fatty liver disease [39]. The increased levels of HDL-C and decreased levels of LDL-C have good effect on regulating lipid metabolism [40], both of which were regulated to normal levels by PTA administration. 

Hyperglycemia and lipotoxicity are the states induced by dyslipidemia, which can lead to oxidative stress via the excess production of ROS and which cause renal injury via the induction of insulin resistance [41]. We observed that PTA provided renal protection against DN by the PTA regulation of BUN, AUG, and ALB, as well as an improvement of pathological morphology in kidney tissue sections. The increased pro-inflammatory cytokines caused by hyperglycemia leads to the glomerular infiltration of monocytes and macrophages, which worsen the progression of DN by modulating the release of pro-inflammatory factors and tissue remodeling cytokines [42]. Based on the screening of inflammatory factors by an antibody array assay and the subsequent detection by ELISA, PTA regulated the levels of ILs, TNF-α, IFN-γ and M-CSF. IL-4 can alter the progression of inflammatory infiltration and interstitial fibrosis in the kidney by altering the susceptibility of immunoglobulin [43]. IL-4 primes also naive T cells into a Th2 response, while IL-12 promotes T cell-producing Th1 cells [44]. Th1 and Th2 cells have been implicated in the development and progression of DN [45]. M-CSF is associated with increased glomerular chemokine expression and glomerular macrophage aggregation [46]. Proinflammatory cytokines including TNF-α and IFN-γ have been recognized as pathogenic mediators that contribute to organ damage [47]. Inflammation in the pathogenesis of DN is tightly associated with the overexpression of TNF-α and the cells that secrete TNF-α during the early and late stages of DN [48]. IL-4 is reported to inhibit the effects of proinflammatory cytokines [49]. Together, these experiments suggest that PTA regulates the levels of inflammatory factors and is involved in renal protection in db/db mice. 

Hyperglycemia increases NF-κB gene expression [50]. NF-κB is at the center of several pathways involved in DN, such as the activation of the renin–angiotensin system, AGE accumulation, and NADPH-dependent oxidative stress [51]. Activated IKKα or IKKβ controlled by AKT helps to phosphorylate IκBs, which further activate NF-κB [52]. Phosphorylated NF-κB transfers to the nucleus, where it controls a large range of genes encoding pro-inflammatory cytokines, such as TNF-α and ILs [53]. Oxidative stress triggers inflammatory reactions like inflammatory cell infiltration and basement membrane thickening by activating NF-κB signaling, and it finally sharpens kidney damage in DN [54]. Several studies have shown that the macromolecules extracted from natural materials, such as *Angelica sinensis* [55], *Grifola frondosa* [56] and *Astragalus membranaceus* [57], can improve DN via the inhibition of the expression of NF-κB. Together, these data suggest that PTA-mediated anti-diabetic nephropathy effects are related to its modulation of NF-κB signaling, as PTA strongly reduces the expression levels of phosphor (P)-NF-κB in the kidney tissues of db/db mice.

While we have performed a preliminary characterization of PTA, the higher-order structure of PTA is still unknown. Future experiments to purify PTA and investigate the relationship between its pharmacological efficacy and structure are necessary to determine the mechanism of its role in protection against DN in T2DM. Moreover, the relationship between the anti-oxidative and anti-inflammatory effects of PTA is still unresolved. 

## 4. Materials and Methods 

### 4.1. Preparation of Polysaccharides from T. albuminosus Fruiting Body

The powder of *T. albuminosus* fruiting body (obtained from Yunnan Property Import and Export Group Co., Ltd., Kunming, Yunnan, China) was extracted in 30-fold double deionized water at 80 °C for 3 h, twice. The collected extractions were concentrated and deproteinized using the Sevag agent (butanol/chloroform, v/v = 1:4). After removing the organic reagent, alcohol was added to the solution to a final concentration of 80%, and the samples were placed at 4 °C overnight. After centrifugation at 10 min, 8000 rpm and 4 °C, the polysaccharides were purified using diethylethanolamine-52 (DEAE−52) (C8930) (Solarbio Biotechnology Co., Ltd, Beijing, China) anion exchange column chromatography (3.6 × 30 cm) under a gradient elution using 0, 0.1 and 0.3 M NaCl solutions at a flow rate of 1.0 mL/min. The collected polysaccharides under the elution with 0.1 M NaCl were entitled PTA, and these were applied to further experiments (Figure 1A).

### 4.2. Characteristic Analysis of PTA

#### 4.2.1. Ultraviolet (UV) Spectra Measurement. 

The UV spectra of PTA were scanned from 200 to 400 nm with an interval of 1 nm and an entrance slit width of 2.0 nm using the UV-3150 UV-Vis-NIR spectrophotometer (Shimadzu Corporation, California, USA).

#### 4.2.2. FT-IR Spectra Analysis. 

One millimeter slices containing 2 mg of PTA and 400 mg of KBr were prepared for FT-IR measurements at a range of 4000–400 cm^−1^ using an infrared spectrometer (IRPrestige-21, Tosoh, Tokyo, Japan). 

#### 4.2.3. Molecular Weight Analysis. 

An LC-10ATvp HPLC system (Shimadzu, Kyoto, Japan) equipped with an Alltech 2000 ES evaporative photodetector (Nicholasville, Kentucky, USA) and a TSK-GEL G4000PWXL column (7.8 × 300 mm, Tosoh, Tokyo, Japan) was used to analyze the molecular weight of PTA. It was operated at 35 °C and eluted by ultrapure water at a flow rate of 0.45 mL/min with an initial injection of 20 μL of PTA sample dissolved in ultrapure water. A standard curve established using dextrans of 1, 5, 12, 25, 50 and 80 kDa (Sigma-Aldrich, St. Louis, Missouri, USA) was used to calculate the molecular weight of PTA. 

#### 4.2.4. Monosaccharides Analysis. 

PTA (20 mg) was hydrolyzed with 4 mol/L H_2_SO_4_ at 105 °C for 8 h and neutralized with BaCO_3_, and the supernatant was obtained after centrifugation at 10 min, 10,000 rpm. The LC−10ATvp HPLC system equipped with a PrevailTM ES carbohydrate analysis column (250 × 4.6 mm) and a 2000ES evaporation light scattering detector (Alltech, Nicholasville, Kentucky, USA) was used to analyze the elements of hydrolyzing samples under a 1.0 mL/min flow rate with 80% acetonitrile. Glc, Rha, Xyl, Gal, Man and L-arabinose (Ara) (Sigma-Aldrich, St. Louis, Missouri, USA) were chosen as monosaccharide standards. 

### 4.3. Experimental Protocol Performed in db/db Mice 

The animal experiments were carried out in accordance with the Guiding Principles of Jilin University Animal Ethics Committee (2017SY0901), which had been approved on 1 September 2017. Eight-week-old male db/db mice (n = 48) and db/m^+^ mice (n = 12) (SCXK (Su) 2015–0001), obtained from the Nanjing Biomedical Research Institute of Nanjing University, Nanjing, Jiangsu, China, were housed in groups of four or five in clear plastic cages and maintained on a 12-h light/dark cycle (lights on 07:00–19:00 h) at 23 ± 1 °C with water and food available ad libitum. The db/db mice were randomly divided into four groups, and they were orally treated with either 10 mL/kg of normal saline as a negative control group (n = 12); 100 mg/kg of Met (Shenzhen Haiwang Pharmaceutical Co. Ltd., Shenzhen, China) serving as a positive control group (n = 12); or 25 mg/kg (n = 12) or 100 mg/kg (n = 12) of PTA as test groups. The db/m+ mice were orally treated with 10 mL/kg of normal saline serving as control group (CTRL) (n = 12). The agent administration was continued for 8 weeks, once per day. Bodyweights were recorded once per day, and fasting blood glucose levels were detected once every week. 

At the beginning of the 9^th^ week, mice were placed in metabolic cages, and the urine volume of each mouse was collected for 24 h. The amount of food and water intake within 24 h of each mouse was recorded. Following this experiment, all mice were fasted for 2 h and then orally administered with 2.0 g/kg of glucose. The concentration of blood glucose was analyzed at 0, 0.5, 2, and 4 h after glucose treatment. The area under the blood glucose curve was calculated according to the following formula. 

Area under the curve (AUC) = 0.5 × (glycemia 0 h + glycemia 0.5 h) × (0.5 h – 0 h) + 0.5 × (glycemia 0.5 h + glycemia 2 h) × (2 h – 0.5 h) +0.5 × (glycemia 2 h + glycemia 4 h) × (4 h – 2 h). 

After the oral glucose tolerance test, all mice were euthanized using carbon dioxide asphyxiation after blood sampling from the caudal vein. Tissues including liver, kidney and pancreas were collected. Tissue was either fixed in 10% formalin or stored at –80 °C.

### 4.4. Histopathological Examination

Fixed tissues were dehydrated in ethyl alcohol (from 70% to 100%), dealcoholized in xylene, embedded in paraffin, and cut into 5 mm sections. The sections were deparaffinized in xylene and rehydrated in ethyl alcohol from 100% to 70% in reverse order. All specimens were stained with hematoxylin and eosin (H and E) to analyze the damage of liver, kidney and pancreas. Images were collected with an inverted CKX41 light microscope (Olympus, Tokyo, Japan). Six slices were prepared and stained parallel in each group.

### 4.5. Antibody Array Assay of Kidney 

An antibody array assay of kidney was process outsourced to Univ-bioscience, Inc. Shanghai, China. A Mouse XL Cytokine Array Kit (ARY028) (R&D Systems, Inc., Minnesota, USA) was used to detect 111 cytokines related to inflammation in the kidney tissues. The protein of each kidney was extracted with ice-cold Cell and Tissue Protein Extraction Reagent (KC-415) (KangChen Bio-tech, Shanghai, China), and concentrations were detected using the butyleyanoacrylate (BCA) protein assay kit (KC-430) (KangChen Bio-tech, Shanghai, China). The array membranes were blocked in a blocking buffer (5% bovine serum albumin (BSA) solution), incubated with samples at 4 °C for 12 h, incubated with diluted biotin-conjugated antibodies at 25 °C for 8 h, incubated with streptavidin-conjugated fluorophores at room temperature for 2 h, and then detected with a chemiluminescence imager (Chemi Scope 6300) (Clinx, Shanghai, China). The analysis was repeated for three times. 

### 4.6. Biochemical Indexes Measurement

The levels of ALB (CK-E91074M), ATP (CK-E93365M), GHbA1c (CK-E20512M), LDL-C (CK-E93032M), HDL-C (CK-E93031M), INS (CK-E43315M), BUN (CK-E92792M) in serum; the levels of IFN-γ (CK-E11382M), IL-2 (CK-E20010M), IL-4 (CK-E42901M), IL-12 (CK-E90205M), TNF-α (CK-E20220M), CAT (CK-E92636M), SOD (CK-E20348M), ROS (CK-E91516M), GSH-Px (CK-E92669M), PKA (CK-E50226M), MMP-9 (CK-E90157M), 6-K-PGF1α (CK-E43000M), LEP (CK-E42895M), and M-CSF (CK-E42892M) in the kidney and the levels of NAG (CK-E20276M) in urine were determined using ELISA kits (Shanghai Yuanye Bio-Technology Co. Ltd., Shanghai, China) according to the manufacturer’s instructions. Twelve samples of each group were prepared for biochemical indexes analysis. 

### 4.7. Western Blotting

Mouse kidney tissues were homogenized with a radio immunoprecipitation assay (RIPA) buffer containing 0.94% 50-mM phenylmethanesulfonyl fluoride (PMSF) (Sigma-Aldrich, St. Louis, Missouri, USA) and 0.97% protease inhibitor cocktail (Sigma-Aldrich, St. Louis, Missouri, USA). The concentration of the collected protein was detected using the BCA kit. Forty micrograms of protein were electrophoresed on a 12% sodium dodecyl sulfate–polyacrylamide gel electrophoresis (SDS-PAGE) and transferred onto a polyvinylidene difluoride (PVDF) membrane (Merck Millipore, Burlington, Massachusetts, USA). After blocking with 5% BSA diluted in a Tris buffer, the membrane was incubated with primary antibodies, including total (T)-NF-κB (ab32536), phosphor (P)-NF-κB (p-NF-κB) (ab86299), P- IKKα+β (S180+S181) (ab55341), T-IκBα (ab32518), T-AKT (ab200195), and P-AKT (ab131443) (Abcam, Cambridge Science Park, UK), T-IKKα+β (bs-7557R) and P-IκBα (Ser36) (bs-18129R) (Bioss Biotechnology Co., Ltd., Beijing, China), and glyceraldehyde-3-phosphate dehydrogenase (GAPDH) (E-AB-20059) (Elabscience Biotechnology Co. Ltd., Wuhan, China) at dilution of 1:2000 at 4 °C overnight. After three washes in a TBS buffer with 0.1% Tween-20, the membranes were then incubated with horseradish peroxidase-conjugated goat anti-rabbit secondary antibodies (E-AB-1003, 1:2000, Elabscience, Wuhan, China) for 4 h at 4°C. A gel imaging system (Tanon Technology Co., Ltd., Shanghai, China) was used to visualize the bands using an electrochemiluminescence (ECL) detection assay (5200) (Tanon Technology Co., Ltd., Shanghai, China). Image J Version 1.8.0 (National Institutes of Health, Bethesda, USA) was used to detect the optical density of bands. Six samples of each group were randomly chosen for western blot analysis. 4.8. Statistical Analysis 

Data were expressed as mean ± SD. Differences were determined by a one-way analysis of variance followed by post-hoc multiple comparisons (Holm–Sidak test) using SPSS 22.0 software (IBM Corporation, New York, USA). Statistical significance was declared for *p* values of less than 0.05. 

## 5. Conclusions 

In this study, we first report that PTA isolated from *T. albuminosus* fruiting bodies shows hypoglycemic, hypolipidemic, and anti-diabetic nephropathy activities. The renal protection of PTA in db/db mice may be related to anti-inflammatory activity via the regulation of NF-κB signaling. The present study provides experimental evidence to support *T. albuminosus* and its polysaccharides as natural food supplements or functional foods for diabetes and DN adjunctive therapy.

## Figures and Tables

**Table 1:** Significantly changed inflammatory factors that were regulated by Met and PTA; authenticated by ELISA.

Kidney (pg/mgprot)	db/m^+^	db/db	db/db		
			Met (0.1g/kg)	PTA (0.025g/kg)	PTA (0.1g/kg)
IL-2	54.8 ± 2.5	72.4 ± 9.3^##^	59.7 ± 6.9*	53.6 ± 3.9**	59.4 ± 7.0*
IL-4	2.7 ± 0.1	2.4 ± 0.1^#^	2.9 ± 0.3**	2.8 ± 0.3**	3.0 ± 0.5**
IL-12	0.80 ± 0.08	1.35 ± 0.14^###^	0.93 ± 0.11***	0.96 ± 0.09***	1.05 ± 0.06**
TNF-α	100.2 ± 6.7	132.7 ± 11.7^##^	116.1 ± 14.0	100.7 ± 10.0**	102.4 ± 4.3**
LEP	0.66 ± 0.05	0.37 ± 0.02^###^	0.65 ± 0.04***	0.65 ± 0.09***	0.66 ± 0.09***
M-CSF	21.2 ± 1.9	26.1 ± 0.6^##^	20.9 ± 1.6**	18.9 ± 2.2***	20.9 ± 1.9**
IFN-γ	2.5 ± 0.3	3.4 ± 0.2^##^	2.4 ± 0.2***	2.2 ± 0.1***	2.7 ± 0.2**

Data are expressed as mean ± SD (n = 12). ^#^ *p* < 0.05, ^##^ *p* < 0.01 and ^###^ *p* < 0.001 vs. db/m^+^ mice, * *p* < 0.05, ** *p* < 0.01 and *** *p* < 0.001 vs. non-treated db/db mice.

## Abbreviations

AKT	Protein Kinase B
ALB	Albumin
Ara	L-Arabinose
ATP	Adenosine Triphosphate
AUC	Area Under the Curve
BCA	Butyleyanoacrylate
BSA	Bovine Serum Albumin
BUN	Blood Urea Nitrogen
CAT	Catalase
CTRL	Control Group
DEAE−52	Diethylethanolamine-52
DM	Diabetes Mellitus
DN	Diabetic Nephropathy
ECL	Electrochemiluminescence
ELISA	Enzyme-Linked Immunosorbent Assay
FT-IR	Fourier Transform Infrared
Gal	D-Galactose
GHbA1c	Glycosylated Hemoglobin A1c
Glc	D-Glucose
H and E	Hematoxylin Eosin
GAPDH	Glyceraldehyde-3-Phosphate Dehydrogenase
GSH-Px	Glutathione Peroxide
HDL-C	High Density Lipid Cholesterol
HPLC	High Performance Liquid Chromatography
IFN-γ	Interferon-Γ
IκBα	Inhibitor Of ΚB Alpha
IKKα+β	Inhibitor Of ΚB Kinase Alpha + Beta
ILs	Interleukins
INS	Insulin
LDL-C	Low Density Lipid Cholesterol
LEP	Leptin
Man	D-Mannose
MCP-1	Monocyte Chemotactic Protein-1
M-CSF	Macrophage Colony-Stimulating Factor
Met	Metformin
MMP-9	Matrix Metalloproteinase-9
NAG	N-Acetyl-Β-D-Glucosidase
NF-κB	Nuclear Factor-ΚB
OGTT	Oral Glucose Tolerance Test
PKA	cAMP-Dependent Protein Kinase
PMSF	Phenylmethanesulfonyl Fluoride
PVDF	Polyvinylidene Difluoride
Rha	L-Rhamnose
RIPA	Radio Immunoprecipitation Assay
ROS	Reactive Oxygen Species
SDS-PAGE	Sodium Dodecyl Sulfate–Polyacrylamide Gel Electrophoresis
SOD	Superoxide Dismutase
T2DM	Type 2 DM
TNF-α	Tumor Necrosis Factor-A
UV	Ultraviolet
Xyl	D-Xylose
6-K-PGF1α	6-Keto Prostaglandin F 1A

## Appendix A

**Table A1:** The effects of Met and PTA on the changes in bodyweight, plasma glucose, and food and water intake of mice.

	Week	db/m+	db/db	db/db		
				Met (100 mg/kg)	PTA (25 mg/kg)	PTA (100 mg/kg)
Body weights (g)	1	20.4 ± 2.2	41.6 ± 2.3^###^	40.6 ± 2.9	40.8 ± 3.0	40.3 ± 2.6
	2	18.4 ± 2.5	42.7 ± 2.6^###^	39.3 ± 3.2*	40.0 ± 3.0*	40.1 ± 2.4*
	3	20.3 ± 2.1	45.4 ± 1.9^###^	42.9 ± 3.0*	41.8 ± 2.8**	42.6 ± 1.8**
	4	21.4 ± 1.1	46.6 ± 2.2^###^	43.8 ± 3.7*	43.8 ± 2.9*	44.3 ± 2.1*
	5	22.1 ± 0.9	48.8 ± 2.9^###^	45.4 ± 2.9*	45.3 ± 2.4**	46.0 ± 2.1*
	6	22.0 ± 0.8	51.5 ± 2.6^###^	45.9 ± 3.1**	45.6 ± 3.2***	46.9 ± 2.1**
	7	23.0 ± 0.8	51.1 ± 3.2^###^	45.3 ± 3.0**	45.9 ± 2.9**	47.8 ± 2.4*
	8	22.7 ± 1.4	51.1 ± 3.1^###^	45.5 ± 1.9***	46.1 ± 2.7**	48.0 ± 2.8*
Plasma glucose (mmol/L)	1	5.0 ± 2.3	17.0 ± 6.2^###^	16.9 ± 6.4	15.6 ± 6.5	17.9 ± 3.5
	2 3	6.6 ± 1.3 6.8 ± 1.6	20.4 ± 3.8^###^ 20.2 ± 3.9^###^	17.8 ± 3.3 18.8 ± 3.2	16.6 ± 3.6* 17.3 ± 2.1	17.9 ± 1.7 17.4 ± 2.3
	4 5	6.7 ± 0.9 6.8 ± 0.8	20.3 ± 3.9^###^ 21.0 ± 3.6^###^	19.0 ± 1.1 17.3 ± 2.9*	18.1 ± 3.2 17.6 ± 3.1	17.2 ± 2.5* 16.7 ± 2.3**
	6 7	6.1 ± 1.0 7.0 ± 1.3	21.2 ± 2.2^###^ 21.7 ± 2.2^###^	17.2 ± 2.3** 17.8 ± 2.6**	18.9 ± 2.3* 18.9 ± 2.7*	16.2 ± 2.7*** 16.1 ± 2.1***
	8	6.7 ± 0.7	23.4 ± 2.3^###^	17.1 ± 2.0***	17.4 ± 3.0***	16.4 ± 2.2***

Data are expressed as mean ± SD (n = 12). ^###^ *p* < 0.001 vs. db/m^+^ mice, * *p* < 0.05, ** *p* < 0.01 and *** *p* < 0.001 vs. non-treated db/db mice.

**Table A2:** All of the detailed parameters of target cytokines decreased or increased among experimental groups.

Coordinate	Target	Fold	
		db/db (vs.db/m+)	db/db+100 mg/kg PTA (vs.db/db)
A 3; A 4	Adiponectin/Acrp30	−24.8%	−0.4%
A 5; A 6	Amphiregulin	18.6%	−6.7%
A 7; A 8	Angiopoietin-1	15.0%	−3.9%
A 9; A 10	Angiopoietin-2	29.8%	3.4%
A 11; A 12	Angiopoietin-like 3	43.6%	−7.8%
A 13; A 14	BAFF/BLyS/TNFSF13B	−1.9%	21.2%
A 15; A 16	C1q R1/CD93	14.1%	9.9%
A 17; A 18	CCL2/JE/MCP-1	−3.6%	30.9%
A 19; A 20	CCL3/CCL4 MIP-1 alpha/beta	0.7%	13.9%
A 21; A 22	CCL5/RANTES	−6.0%	−12.3%
B 3; B 4	CCL6/C10	31.9%	−17.4%
B 5; B 6	CCL11/Eotaxin	23.3%	−5.9%
B 7; B 8	CCL12/MCP-5	12.7%	−9.8%
B 9; B 10	CCL17/TARC	17.1%	−11.0%
B 11; B 12	CCL19/MIP-3 beta	3.2%	1.9%
B 13; B 14	CCL20/MIP-3 alpha	−12.6%	13.9%
B 15; B 16	CCL21/6Ckine	3.2%	0.9%
B 17; B 18	CCL22/MDC	−3.4%	20.2%
B 19; B 20	CD14	5.5%	11.1%
B 21; B 22	CD40/TNFRSF5	29.0%	−11.6%
C 3; C 4	CD160	8.9%	5.7%
C 5; C 6	Chemerin	20.7%	−5.1%
C 7; C 8	Chitinase 3-like 1	187.7%	−60.1%
C 9; C 10	Coagulation Factor III/ Tissue Factor	−4.9%	−1.2%
C 11; C 12	Complement Component C5/C5a	−7.6%	1.7%
C 13; C 14	Complement Factor D	−60.4%	17.6%
C 15; C 16	C-Reactive Protein/CRP	44.5%	−8.6%
C 17; C 18	CX3CL1/Fractalkine	−20.9%	6.8%
C 19; C 20	CXCL1/KC	2.4%	7.6%
C 21; C 22	CXCL2/MIP-2	11.3%	6.9%
D 1; D 2	CXCL9/MIG	4.8%	−25.2%
D 3; D 4	CXCL10/IP-10	8.7%	−20.1%
D 5; D 6	CXCL11/I-TAC	−0.3%	−6.8%
D 7; D 8	CXCL13/BLC/BCA-1	31.4%	−28.2%
D 9; D 10	CXCL16	20.5%	−6.3%
D 11; D 12	Cystatin C	−1.0%	−6.7%
D 13; D 14	DKK-1	−8.5%	16.9%
D 15; D 16	DPPIV/CD26	−13.1%	3.0%
D 17; D 18	EGF	−4.5%	−3.2%
D 19; D 20	Endoglin/CD105	−6.2%	−2.3%
D 21; D 22	Endostatin	3.2%	−9.9%
D 23; D 24	Fetuin A/AHSG	−11.2%	−16.1%
E 1; E 2	FGF acidic	−2.6%	−2.9%
E 3; E 4	FGF-21	−7.4%	−5.0%
E 5; E 6	Flt-3 Ligand	−6.4%	1.8%
E 7; E 8	Gas 6	9.7%	−7.1%
E 9; E 10	G-CSF	2.7%	7.0%
E 11; E 12	GDF-15	4.5%	18.4%
E 13; E 14	GM-CSF	−8.8%	19.0%
E 15; E 16	HGF	−16.4%	26.5%
E 17; E 18	ICAM-1/CD54	−10.9%	1.1%
E 19; E 20	IFN-gamma	−14.9%	45.9%
E 21; E 22	IGFBP-1	32.6%	−2.9%
E 23; E 24	IGFBP-2	−53.4%	−2.0%
F 1; F 2	IGFBP-3	−3.0%	4.9%
F 3; F 4	IGFBP-5	−4.3%	4.5%
F 5; F 6	IGFBP-6	−11.0%	−5.2%
F 7; F 8	IL-1 alpha/IL-1F1	5.0%	−1.8%
F 9; F 10	IL-1 beta/ IL-1F2	17.8%	−10.7%
F 11; F 12	IL-1ra/IL-1F3	58.9%	−31.1%
F 13; F 14	IL-2	19.2%	0.4%
F 15; F 16	IL-3	−9.8%	12.1%
F 17; F 18	IL-4	−25.4%	18.2%
F 19; F 20	IL-5	−0.7%	−10.7%
F 21; F 22	IL-6	32.4%	0.7%
F 23; F 24	IL-7	4.4%	−8.8%
G 1; G 2	IL-10	6.0%	3.9%
G 3; G 4	IL-11	−4.1%	8.2%
G 5; G 6	IL-12p40	3.6%	6.4%
G 7; G 8	IL-13	8.6%	−4.4%
G 9; G 10	IL-15	17.8%	−12.5%
G 11; G 12	IL-17A	22.4%	12.9%
G 13; G 14	IL-22	5.3%	−2.2%
G 15; G 16	IL-23	0.3%	3.1%
G 17; G 18	IL-27p28	−8.5%	0.7%
G 19; G 20	IL-28	−10.8%	−1.9%
G 21; G 22	IL-33	20.7%	26.1%
G 23; G 24	LDL R	9.6%	−2.5%
H 1; H 2	Leptin	156.9%	−14.2%
H 3; H 4	LIF	11.9%	−17.0%
H 5; H 6	Lipocalin-2/NGAL	97.3%	−34.9%
H 7; H 8	LIX	31.5%	−29.8%
H 9; H 10	M-CSF	21.6%	−11.2%
H 11; H 12	MMP-2	20.1%	−18.8%
H 13; H 14	MMP-3	24.6%	−25.1%
H 15; H 16	MMP-9	64.0%	−33.7%
H 17; H 18	Myeloperoxidase	83.5%	−49.4%
H 19; H 20	Osteopontin (OPN	62.5%	−17.2%
H 21; H 22	Osteoprotegerin/ TNFRSF11B	13.2%	−14.3%
H 23; H 24	PD-ECGF/ Thymidine phosphorylase	13.7%	−2.1%
I 1; I 2	PDGF-BB	21.4%	−36.0%
I 3; I 4	Pentraxin 2/SAP	14.3%	−15.2%
I 5; I 6	Pentraxin 3/ TSG-14	21.5%	−17.7%
I 7; I 8	Periostin/OSF-2	42.9%	−16.2%
I 9; I 10	Pref-1/DLK-1/FA1	28.1%	−19.2%
I 11; I 12	Proliferin	16.8%	−3.7%
I 13; I 14	Proprotein Convertase 9/ PCSK9	3.5%	−26.6%
I 15; I 16	RAGE	16.6%	−16.2%
I 17; I 18	RBP4	15.4%	−28.8%
I 19; I 20	Reg3G	13.9%	−11.2%
I 21; I 22	Resistin	−39.9%	−0.5%
J 3; J 4	E-Selectin/CD62E	7.8%	−23.9%
J 5; J 6	P-Selectin/CD62P	−14.3%	−7.8%
J 7; J 8	Serpin E1/PAI-1	20.4%	−27.4%
J 9; J 10	Serpin F1/PEDF	4.1%	−29.5%
J 11; J 12	Thrombopoietin	17.0%	−12.3%
J 13; J 14	TIM-1/KIM-1/ HAVCR	388.0%	−62.9%
J 15; J 16	TNF-alpha	32.0%	−24.4%
J 17; J 18	VCAM-1/CD106	28.3%	−30.9%
J 19; J 20	VEGF	−3.9%	−11.7%
J 21; J 22	WISP-1/CCN4	−2.2%	−18.9%
